# Supplementary material for: Perinatal Fat-Diets Increased Angiotensin II-Mediated Ca2+ through PKC-L-Type Calcium Channel Axis in Resistance Arteries via Agtr1a-Prkcb Gene Methylation
Source: Nutrients. 2023 Jan 3;15(1):245. doi: 10.3390/nu15010245 (PMC9824013; doi:10.3390/nu15010245)
Supplement: Supplementary file 1 [file nutrients-15-00245-s001.zip › nutrients-2110248-supplementary.pdf]

Table S1. The methylation of CpG locus at *Agtr1a* and *Prkcb* gene promoter. The data was expressed as mean ± SEM. CON control group, MHF maternal high fat diet group. *P < 0.05

**Gene**	**Number**	**Position**	**CON**	**MHF**	**P value**	
Agtr1a	1	29	0.01757±0.004964	0.01505±0.000562	0.628	
	2	38	0.01870±0.005351	0.02121±0.003137	0.697	
	3	54	0.01628±0.003589	0.01626±0.001662	0.996	
	4	60	0.02066±0.003756	0.01671±0.001150	0.343	
	5	65	0.01731±0.002828	0.01206±0.003130	0.249	
	6	111	0.02203±0.003391	0.02201±0.002948	0.996	
	7	116	0.02266±0.003799	0.01092±0.001415	0.02	*
	8	125	0.02198±0.005382	0.02146±0.002313	0.933	
	9	131	0.01915±0.003312	0.01468±0.002536	0.315	
	10	140	0.02392±0.004576	0.02647±0.002769	0.646	
	11	147	0.02458±0.002385	0.02019±0.002291	0.221	
	12	154	0.01925±0.004154	0.01587±0.002898	0.523	
	13	177	0.01708±0.003677	0.01632±0.001561	0.853	
	14	191	0.01304±0.002708	0.01054±0.002460	0.514	
	15	64	0.02459±0.003512	0.02628±0.002273	0.698	
	16	91	0.04522±0.003752	0.02966±0.003492	0.016	*
	17	117	0.02462±0.004641	0.01795±0.001863	0.219	
	18	139	0.04431±0.005855	0.02939±0.002574	0.048	*
	19	154	0.04213±0.005353	0.03052±0.003737	0.113	
	20	177	0.02374±0.006598	0.01405±0.002175	0.201	
	21	189	0.02102±0.004500	0.01462±0.002494	0.249	
	22	197	0.01701±0.004826	0.00958±0.001597	0.182	
	23	215	0.02282±0.005352	0.01723±0.002801	0.381	
Prkcb	1	22	0.01242±0.001198	0.01212±0.002027	0.904	
	2	24	0.00932±0.001923	0.01051±0.000661	0.575	
	3	34	0.01066±0.001180	0.01348±0.001502	0.178	
	4	38	0.01783±0.001208	0.02107±0.002518	0.279	
	5	47	0.02055±0.002825	0.02091±0.000519	0.902	
	6	51	0.01312±0.001125	0.01304±0.002213	0.973	
	7	58	0.02307±0.002166	0.02453±0.001291	0.579	
	8	66	0.01601±0.001887	0.01639±0.002747	0.912	
	9	68	0.01231±0.001534	0.00836±0.001349	0.089	
	10	79	0.01418±0.001670	0.01819±0.001187	0.086	
	11	81	0.01325±0.001827	0.01218±0.001750	0.684	
	12	84	0.01851±0.000918	0.01849±0.001177	0.987	
	13	93	0.01736±0.001560	0.01652±0.002033	0.751	
	14	96	0.01158±0.002350	0.01393±0.001043	0.387	
	15	100	0.01655±0.001848	0.01780±0.002106	0.666	

	16	102	0.01461±0.001213	0.01178±0.002323	0.312	
	17	104	0.01364±0.001297	0.01349±0.001349	0.936	
	18	106	0.01573±0.000968	0.01595±0.001740	0.915	
	19	108	0.01260±0.002167	0.01144±0.001698	0.686	
	20	126	0.01367±0.001241	0.01254±0.001675	0.602	
	21	140	0.01523±0.002214	0.01506±0.000559	0.944	
	22	164	0.01466±0.000567	0.01598±0.001129	0.328	
	23	31	0.01545±0.000696	0.01581±0.002281	0.885	
	24	48	0.01521±0.002201	0.01553±0.001059	0.9	
	25	56	0.01075±0.000992	0.01255±0.002184	0.475	
	26	65	0.01160±0.001405	0.01404±0.000809	0.171	
	27	78	0.01022±0.001917	0.00975±0.000761	0.825	
	28	82	0.01120±0.001812	0.01390±0.000944	0.223	
	29	86	0.01343±0.001924	0.00999±0.002311	0.286	
	30	111	0.01969±0.001905	0.01331±0.001140	0.021	*
	31	118	0.01129±0.000649	0.01294±0.001656	0.381	
	32	136	0.02156±0.002697	0.02143±0.001057	0.964	

CON: Control group; MHF: maternal high fat diet group

Table S2. Methylated CpG sites at *Agtr1a* and *Prkcb* gene promoter

Target	Number	Position	Chromosome	Genome Position	Distance to TSS
Agtr1a	1	29	chr17	35957894	183
Agtr1a	2	38	chr17	35957903	174
Agtr1a	3	54	chr17	35957919	158
Agtr1a	4	60	chr17	35957925	152
Agtr1a	5	65	chr17	35957930	147
Agtr1a	6	111	chr17	35957976	101
Agtr1a	7	116	chr17	35957981	96
Agtr1a	8	125	chr17	35957990	87
Agtr1a	9	131	chr17	35957996	81
Agtr1a	10	140	chr17	35958005	72
Agtr1a	11	147	chr17	35958012	65
Agtr1a	12	154	chr17	35958019	58
Agtr1a	13	177	chr17	35958042	35
Agtr1a	14	191	chr17	35958056	21
Agtr1a	15	64	chr17	35957703	374
Agtr1a	16	91	chr17	35957730	347
Agtr1a	17	117	chr17	35957756	321
Agtr1a	18	139	chr17	35957778	299
Agtr1a	19	154	chr17	35957793	284
Agtr1a	20	177	chr17	35957816	261

Agtr1a	21	189	chr17	35957828	249
Agtr1a	22	197	chr17	35957836	241
Agtr1a	23	215	chr17	35957854	223
Prkcb	1	22	chr1	192233826	47
Prkcb	2	24	chr1	192233824	45
Prkcb	3	34	chr1	192233814	35
Prkcb	4	38	chr1	192233810	31
Prkcb	5	47	chr1	192233801	22
Prkcb	6	51	chr1	192233797	18
Prkcb	7	58	chr1	192233790	11
Prkcb	8	66	chr1	192233782	3
Prkcb	9	68	chr1	192233780	1
Prkcb	10	79	chr1	192233769	-10
Prkcb	11	81	chr1	192233767	-12
Prkcb	12	84	chr1	192233764	-15
Prkcb	13	93	chr1	192233755	-24
Prkcb	14	96	chr1	192233752	-27
Prkcb	15	100	chr1	192233748	-31
Prkcb	16	102	chr1	192233746	-33
Prkcb	17	104	chr1	192233744	-35
Prkcb	18	106	chr1	192233742	-37
Prkcb	19	108	chr1	192233740	-39
Prkcb	20	126	chr1	192233722	-57
Prkcb	21	140	chr1	192233708	-71
Prkcb	22	164	chr1	192233684	-95
Prkcb	23	31	chr1	192234357	578
Prkcb	24	48	chr1	192234340	561
Prkcb	25	56	chr1	192234332	553
Prkcb	26	65	chr1	192234323	544
Prkcb	27	78	chr1	192234310	531
Prkcb	28	82	chr1	192234306	527
Prkcb	29	86	chr1	192234302	523
Prkcb	30	111	chr1	192234277	498
Prkcb	31	118	chr1	192234270	491
Prkcb	32	136	chr1	192234252	473

Table S3. The primers used in this study.

**qRT-PCR primers**	**Nucleotide Sequence (5'to 3')**

Gapdh-Forward	AACGACCCCTTCATTGACCT
Gapdh-Reverse	CCCCATTTGATGTTAGCGGG
Agtr1a-Forward	CTTCTCAATCTCGCCTTGGC
Agtr1a-Reverse	AAGGAACACACTGGCGTAGA
Agtr1b-Forward	GGTTCAAAGCCTGCAAGTGA
Agtr1b-Reverse	CGGTTAACAGTGGCTTTGCT
Agtr2-Forward	TGGCTTCCCTTCCATGTTCT
Agtr2-Reverse	TCTCTCTCTTGCCTTGGAGC
Prkca-Forward	AATTCATCGCCCGCTTCTTC
Prkca-Reverse	CACAGAAGGTAGGGCTTCCA
Prkcb-Forward	GGGCATCATTTACCGTGACC

Prkcb-Reverse	GGGGCAATGTAGTCTGGAGT
Prkcd-Forward	CCACCTTCTGTTCTGTGTGC
Prkcd-Reverse	CCTTGAATCGGTGAGGCATG
Prkce-Forward	AGCCTCGTCGTCTACTGATG
Prkce-Reverse	ATCCTTACCCTTGAGCTCGG
**Bisulfite sequencing PCR**	**Nucleotide Sequence (5'to 3')**
Agtr1a-1-Forward	TTTGGATTTAGATGTGGTGATAAGG
Agtr1a-1-Reverse	AAAATACCTAACTACTAACCTCCAAACAA
Agtr1a-2-Forward	TAAAAGTAYGGAGGAAAATTGGTTTGT

Agtr1a-2-Reverse	CACCACATCTAAATCCAAAATACTAAAC
Prkcb-1-Forward	GGGGTTGTAAGTTYGGAGAGG
Prkcb-1-Reverse	TCCCTCCCCCRCAACTAAA
Prkcb-2-Forward	TTGTTTTGTYGAGGAGGGATAGTT
Prkcb-2-Reverse	CCAAATAACCAATCCCTCAAATAC

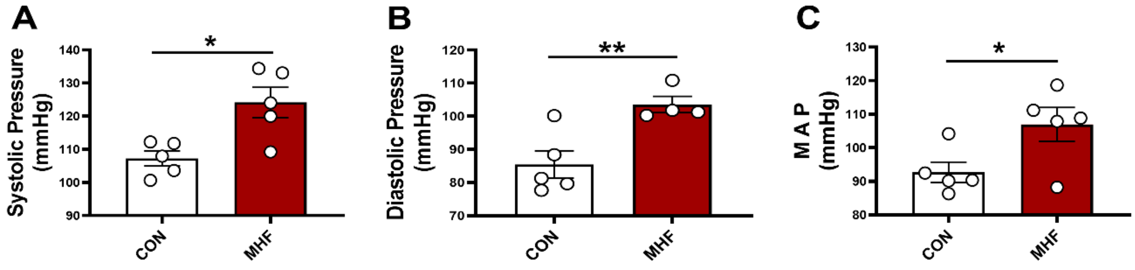

Figure S1. Systolic pressure, diastolic pressure, and mean arterial pressure

A-C, Systolic pressure, diastolic pressure, and mean arterial pressure (MAP) at baseline level in the control (CON) and maternal high fat diets (MHF) groups (N=4-5).

N, number of adult male offspring from different litters of each group. Data were presented as mean±SEM and analyzed by Students-t test. * P < 0.05, ** P < 0.01

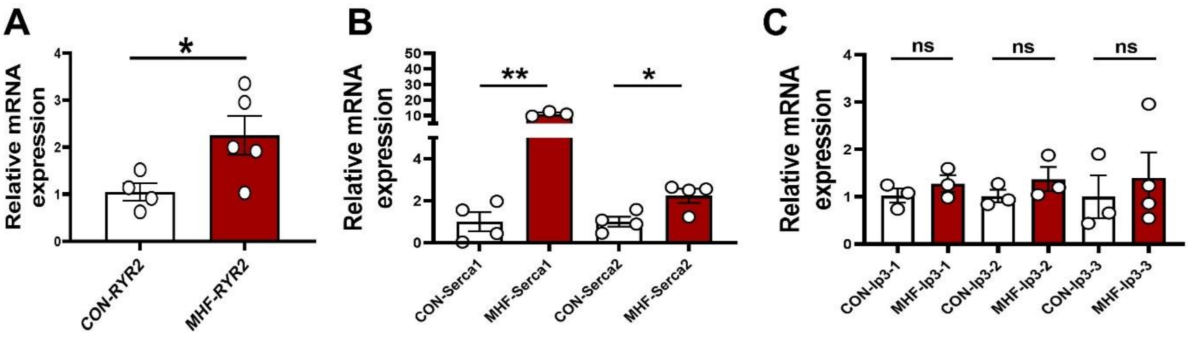

Figure S2. mRNA analysis between the two groups

A-C, Expressions of mRNA in RYR2 (a subtype of Ryanodine receptors), Serca1, Serca2, and IP3 subtype1-3 in adult offspring MA determined by qRT-PCR (N=3, n=3-5). N, number of adult male offspring from different litters of each group; n, number of MA rings. Data were presented as mean±SEM and analyzed by Students- t test. * P < 0.05, ** P < 0.01

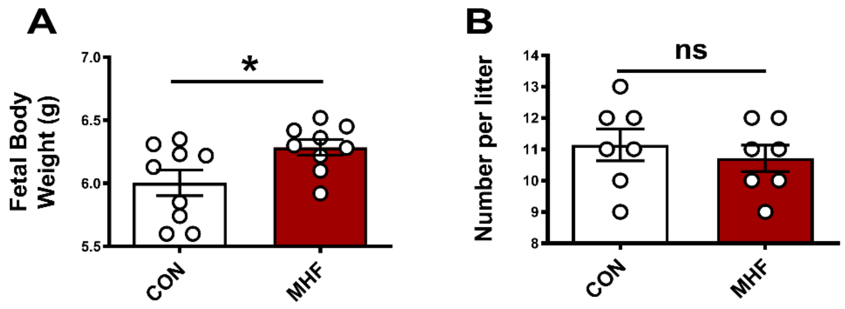

Figure S3. Fetal weights and number per litter

A, The effect of maternal normal diet and high fat diet on offspring fetal body weight (N=9 each group). B, Fetal number per litter (N=7 each group). N, number of adult male offspring from different litters of each group. Data were presented as mean ± SEM and analyzed by Student-t test. *P<0.05

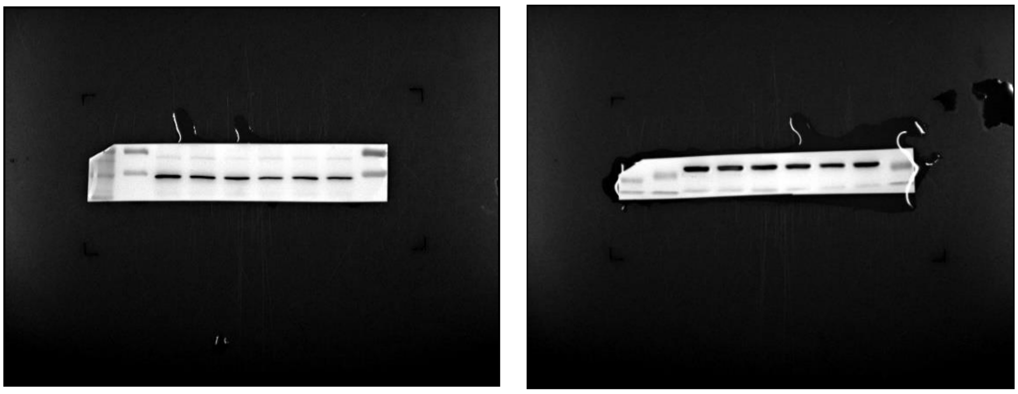

Figure S4

The representative protein images of AT1R (left) and Gapdh (right). The three bands on the left were the MHF group, three bands on the right were the CON group.

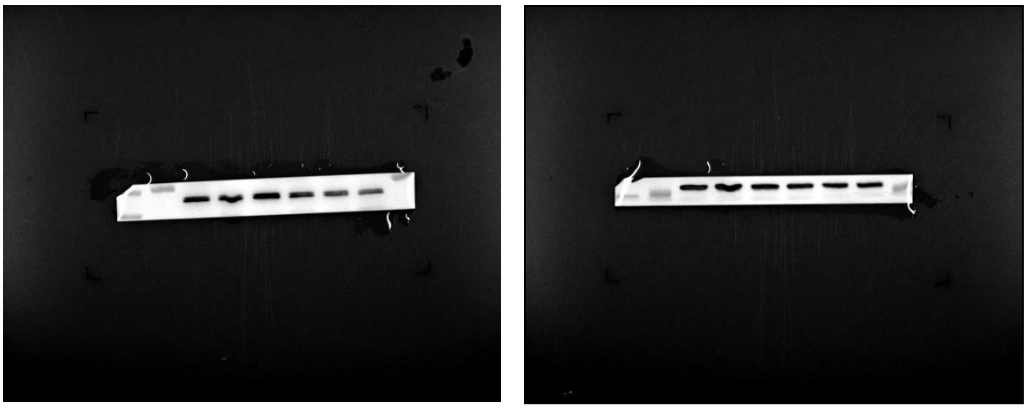

Figure S5

The representative protein images of AT2R (left) and Gapdh (right). The three bands on the left were the MHF group, three bands on the right were the CON group.

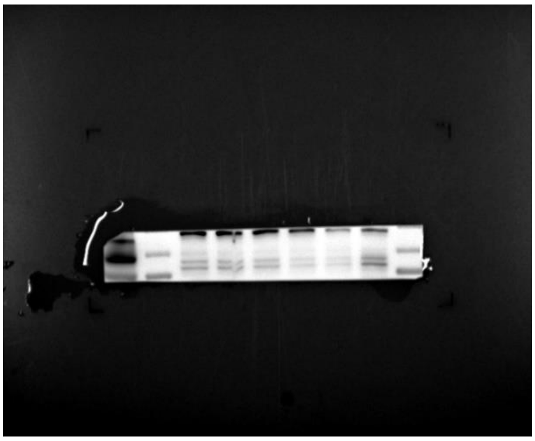

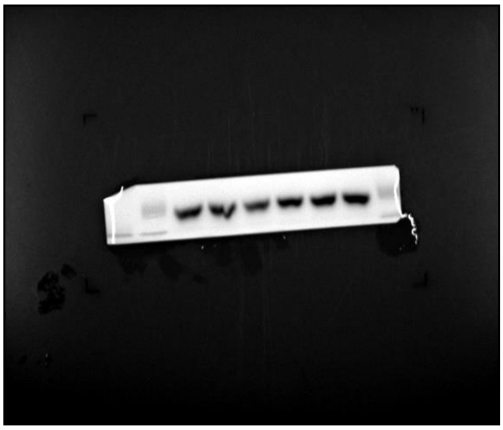

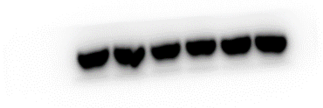

Figure S6

The representative protein images of PKCβ (upper left) and Gapdh (upper right and below). The three bands on the left were the MHF group, three bands on the right were the CON group.

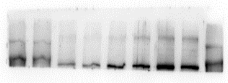

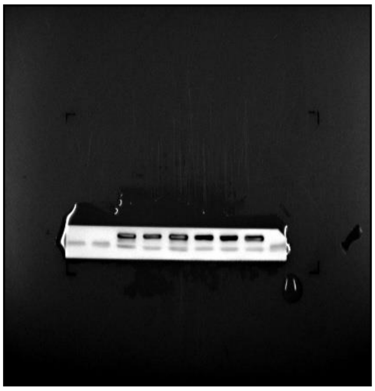

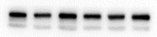

Figure S7

The representative protein images of Cacna1c (left figure) and Gapdh (middle and right figures). The three bands on the left were the CON group, three bands on the right were the MHF group.

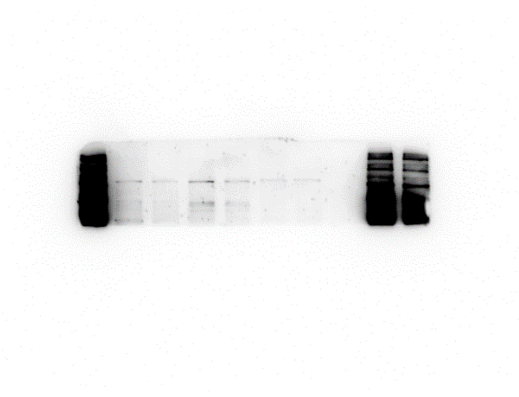

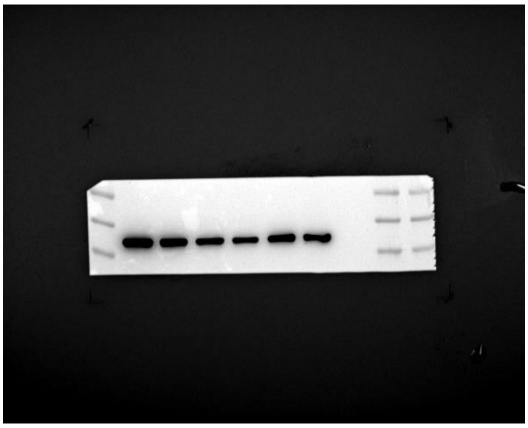

Figure S8

The representative protein images of PKCβ (cells of CON group) (right four bands) and Gapdh (right four bands). The two bands on the left were the vehicle group and the two bands on the right are the protein bands with losartan.

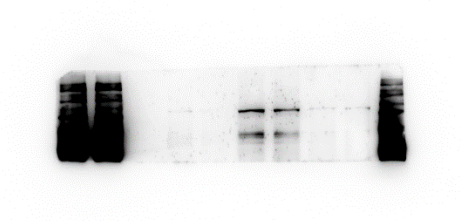

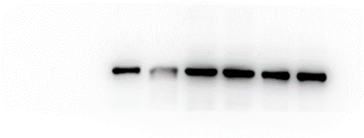

Figure S9

The representative protein images of PKCβ (cells of MHF group) (right four bands) and Gapdh (right four bands). The two bands on the left were the vehicle group and the two bands on the right are the protein bands with losartan.
